# Effects of nano-copper on maize yield and inflammatory response in mice

**DOI:** 10.22038/ijbms.2019.35787.8526

**Published:** 2019-07

**Authors:** Le Thi Thu Hien, Phi Thi Thu Trang, Pham Cam Phuong, Pham Thi Tam, Nguyen Thi Xuan

**Affiliations:** 1 Institute of Genome Research, Vietnam Academy of Science and Technology, 18 Hoang Quoc Viet, Cau Giay, Hanoi, Vietnam; 2 Graduate University of Science and Technology, Vietnam Academy of Science and Technology, 18 Hoang Quoc Viet, Cau Giay, Hanoi, Vietnam; 3 Nuclear Medicine and Oncology Center, Bach Mai Hospital, 78 Giai Phong, Hanoi, Vietnam; 4 Hanoi Open University, 101 Nguyen Hien, Hai Ba Trung, Hanoi, Vietnam

**Keywords:** ALT, AST, Copper, Leukocytes, Maize

## Abstract

**Objective(s):**

Copper (Cu) is an essential dietary supplement in animal feeds, which plays an important role in maintaining the balance of all living organisms. Copper nanoparticles (nCu) participate in catalysing activities of multiple antioxidant/defensive enzymes and exerts pro-inflammatory and pro-apoptotic effects on systemic organs and tissues. The present study explored whether nCu affects maize growth and yield and grain mineral nutrients as well as physiological functions in mice.

**Materials and Methods:**

Maize seeds were treated with nCu (20 mg/kg and 1000 mg/kg dry weight (DW)) and their grain productions were used for mouse feed. For testing of autoimmune response, mice were treated with nCu at concentration of 2 mg/l and 1000 mg/l and ultimately serum biochemical indicators, numbers and activation of immune cells infiltrated in mouse spleens were examined.

**Results:**

Treatment of maize seeds with nCu at dose of 20 mg/kg DW, but not 1000 mg/kg DW enhanced germination rate, plant growth and grain yield as well as grain mineral nutrients as compared to control group. Importantly, administration of mice with 1000 mg/l nCu resulted in their morphological change due to excessive accumulation of nCu in liver and blood, leading to inflammatory responses involved in upregulated expression of serum biochemical indicators of liver and kidney as well as increased infiltration and activation of splenic immune cells.

**Conclusion:**

nCu concentration at 20 mg/kg DW facilitated the morphological and functional development of maize plants, whose production was safe to feed mice.

## Introduction

Copper (Cu) is an essential dietary supplement in animal feeds, which plays an important role in maintaining the balance of all living organisms (1). An accumulation or deficiency of this element may result in significant side effects (2-5) since it participates in regulation of gene expression of oxidation enzymes and cell injury by taking part in oxidation of membrane thiol groups to disulphides (6). As reported, content of Cu in drinking water supply should be less than 2 mg/l (7). Ingested Cu is mainly stored in the liver and mobilized into the circulation and then is distributed to every tissue in the body (1). Cu deficiency is a risk factor of slowed growth, anaemia, impaired bone tissue formation, and cardiac fibrosis (6) as well as chronic lung inflammation (3). By contrast, excessive storage of Cu also induces inflammation-mediated immune response in multiple systemic organs including brain (4), liver and kidney (1, 2, 5). 

Copper nanoparticles (nCu) are used as a dietary supplement due to large surface area facilitating special catalytic activity of multiple enzymes of antioxidant defense system and absorption of mineral elements that are not observed by Cu microparticles (8). The toxicity of nCu depends on its shapes as spherical particles are found to be the most toxic and followed by rod and polygonal shaped particles, and rough surface of nCu may cause fast decomposition of the particles (9). 

Recent studies have revealed that excess Cu exerts pro-inflammatory effects by stimulating receptor-mediated signalling molecules such as nuclear factor-kappa B (NF-κB) (10), resulting in infiltration and activation of immune cells in inflamed organs (11). The recruited innate immune cells to the site of injury are again exposed to Cu to become activated and subsequently transmit activation signals to T lymphocytes, the key players of adaptive immunity (12). Following activation, the infiltrating immune cells produce a large amount of mediators and inflammatory cytokines causing clinical damage to living tissues (11, 12). Liver injury is characterized by accelerated serum levels of alanine transaminase (ALT) and aspartate transaminase (AST) (12), and impaired kidney function is reflected by a significant elevation of serum urea and creatinine concentrations (2, 13). After activation, the immune cells undergo programmed cell death characterized by extracellular exposure of phosphatidylserine (PS), various caspase activities and DNA fragmentation (14). Several recent studies reported that the pro-apoptotic property of Cu is mediated through oxidative stress and DNA damage in different cell types (15, 16). Because the accumulation of Cu in blood circulation and various organs of mammals can cause growth disorders (2, 17, 18), the biological safety issues of Cu content provided for animal feed is needed to be investigated. 

Besides, Cu is required for plant growth and development by regulating biological processes including catalyzing redox reactions, the photosynthetic and respiratory electron transport chain and ATP synthesis (19, 20), therefore Cu influences plant morphological and physiological responses such as seed germination, plant height, grain yield and mineral nutrients (19, 21). In addition, Cu exerts plant antifungal effect by inducing antioxidant/defensive enzyme activity in different plant species including maize (22) and tomato (21). A study by Adrees *et al*. reported that addition of Cu at concentration below 5 mg/kg dry weight (DW) shows a reduction in plant growth (19). In contrast, excessive Cu content also causes impairment of plant metabolic functions including enhanced generation of reactive oxygen species (ROS), oxidative damages and genetic mutations (19). Moreover, excessive Cu contributes to plant tolerance by inducing an increased expression of enzymes against ROS and binding of this metal to plant cell wall to stimulate secretion of toxins (20). The maximum permissible level of Cu for plant nutrition is different from one to another and depends on the growth stage of plants (20). Recent studies on metal nanoparticles have shown that nCu is to facilitate the absorption of water from extracellular matrix (23), leading to an enhanced synthesis of nutrients by passive protein channels/pumps in plants.

In this investigation, we performed experiments to determine the effects of nCu on the growth characteristics of maize plants and the biological safety of maize grains obtained from nCu-treated seeds for mouse feed as well as analyse the effects of nCu on systemic inflammatory response including mouse liver and kidney damages and infiltration, activation and apoptotic cell death of splenic immune cells. 

## Materials and Methods

***Maize seed treatment, cultivation and analysis***

The size of nCu particles (20-30 nm) was confirmed by transmission electron microscopy before usage, and nCu suspension was prepared as previously described (23, 24). Maize seeds were treated with or without nCu (20 mg/kg DW or 1000 mg/kg DW) for 24 hrs before sowing. After that, maize field trials were conducted on an experimental station of Maize Research Institute, Hanoi, Vietnam in randomized complete block design with three replications. In each replication, seedlings were grown in a plot (size 49 m^2^, where row to row and plant to plant spaces were 0.7 m and 0.25 m, respectively) having seven rows (each 10 m). The germination of maize seeds was evaluated after 7 days of growth.

Maize plants were harvested at reproductive maturity. The grains were air-dried under the shade for one day and followed by oven-drying at 65 ± 2^o^C to a constant weight. They were then weighed to determine the grain DWs. Concentrations of mineral elements in the maize grains were calculated according to atomic absorption spectroscopy method (25).

***Mice*** ***and experimental design***

BALB/c mice were purchased from Taconic Farms (Hudson, NY, USA) and housed in a specific pathogen-free facility at Institute of Genome Research. Mice were housed in polypropylene cages and maintained in an animal house at 20 ± 5 °C and 12 hr light/dark cycle. 

The experiment was carried out on fourty BALB/c mice of 8 weeks old and 15-17 g weight by dividing them into 5 experimental groups (each group had four male and four female mice). They were fed diets and drinks as follows: (1) group 1: a basal diet and drink (control); (2) group 2: a grain maize diet derived from 20 mg/kg DW nCu-treated seeds (nCu-20); (3) group 3: a grain maize diet derived from 1000 mg/kg DW nCu-treated seeds (nCu-1000); (4) group 4: a basal diet and drink containing 2 mg nCu/l (2 mg nCu/l-treated mice), and (5) group 5: a basal diet and drink containing 1000 mg nCu/l (1000 mg nCu/l-treated mice). The drinking water was changed every 12 hr. The duration of this experiment was 8 weeks; the body weight (BW) gain of each mouse was recorded before and every two weeks after the administration. All experiments were repeated twice. At the end of the treatment, mice were sacrificed and spleens, livers and serum of the five mouse groups were collected for the subsequent experiments.

***Determination of Cu content in liver and serum***

Cu content in liver tissue and serum of mice was measured by using an Agilent 7500 inductively coupled plasma-mass spectrometer (ICP-MS, Aligent Technologies). The samples were assigned the same weights and digested by the addition of nitric acid (HNO_3 _67%, MOS grade) and hydrogen peroxide (H_2_O_2_ 30%, MOS grade) in glass mini-backers. Two days later, each acid digestion was heated at 80°C for evaporation, 2% nitric acid was added to 10 ml (Cu measurement in liver tissue) or 1 ml (Cu measurement in serum) as the final volume. Calibration plots of standards of Cu were obtained by injecting a series of standard solutions (10, 50, 100, 500 ng/ml in 2% HNO_3_, flow rate 1.0 ml/min). Then, the resulting solution was injected into the ICP-MS system.

***Biochemical assay of serum***

After 8 weeks of treatment, blood samples were obtained from ophthalmic veins (about 0.7-1 ml each mouse). Then, the blood samples were centrifuged at 4000 rpm for 10 min to collect serum. The serum levels of biochemical indicators including ALT, AST, creatinine and urea concentrations were assayed by using an automatic biochemical analyser (Hitachi 7180 Biochemistry Automatic Analyser).

***Flow cytometry***

Numbers and activation of splenic leukocytes were analysed by flowcytometry (FACSAria Fusion, BD Biosciences) as described (12). Splenic cells (10^6^) were incubated in 100 µl FACS buffer (phosphate buffered saline (PBS) plus 0.1% FCS) containing fluorochrome-conjugated antibodies at a concentration of 10 µg/ml. The cells were stained with fluorochrome-coupled antibodies (eBioscience, USA) to CD45, CD3, CD4, CD8, CD19, CD11b, CD11c, F4/80, I-A/I-E and CD69. After incubating with the Abs for 60 min at 4 ^o^C, cells were washed twice and resuspended in FACS buffer. A total of 2 x 10^4^ cells were analysed with flowcytometry.

***Phosphatidylserine translocation and PI incorporation***

To discriminate necrotic/late apoptotic from early apoptotic cells, the presence of PS on the outer surface of the apoptotic cells was detected from fluorescein isothiocyanate (FITC)-conjugated annexin V binding to PS at the cell surface (14), and necrosis/late apoptosis was assessed from the amount of propidium iodide (PI)-positive cells. Briefly, 10^6^ splenic leukocytes were harvested and washed twice with annexin washing buffer (AWB, 10 mM Hepes/NaOH, pH 7.4, 140 mM NaCl, 5 mM CaCl_2_). The cell pellet was resuspended in 100 µl of annexin-V/PI labelling solution (eBioscience, USA), and incubated for 15 min at room temperature. After washing with AWB, CD45-positive lymphocytes were analysed by flowcytometry.

***Statistics***

Data are provided as means ± SEM, and *n* represents the number of independent experiments. All data were tested for significance using Student’s unpaired two-tailed *t*-test or ANOVA, and only results with *P*<0.05 were considered statistically significant.

## Results

***Effect of nCu on seed germination, plant growth,*** ***grain mineral*** ***concentrations*** ***and yield of maize***

**Figure 1:**
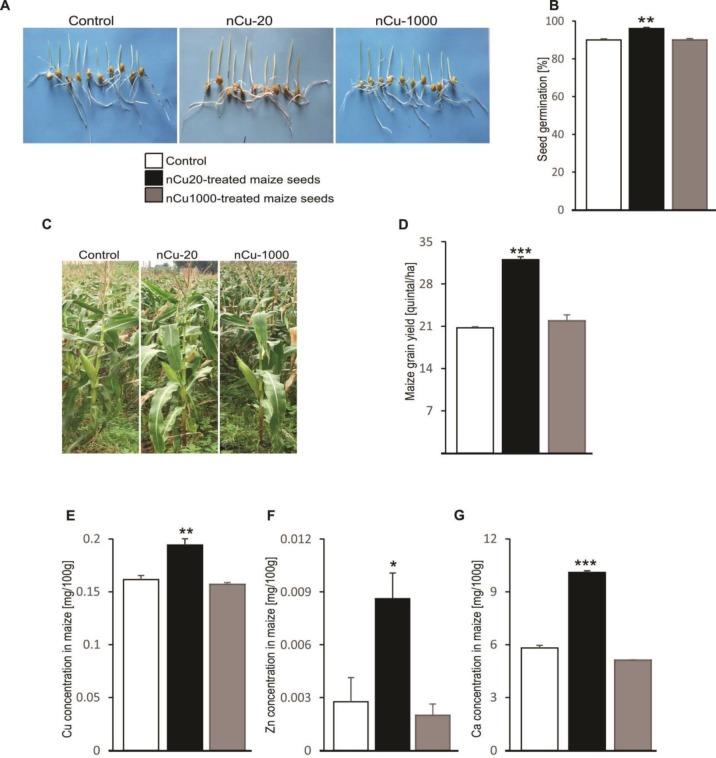
Effect of nano-copper on seed germination, plant growth, grain mineral concentrations and yield of maize

Choudhary *et al*. reported that Cu-chitosan nanoparticle contributes to seed germination and the growth of maize plants (22). In the agreement, maize seed treatment with a 20 mg/kg DW dose of nCu significantly enhanced germination rate after 7 days in the pot experiment (Figure 1A-B), plant growth after 90 days in the experimental station (Figure 1C) and maize yield (Figure 1D). In addition, 20 mg/kg DW nCu treatment upregulated concentration of grain mineral nutrients including Cu (Figure 1E), zinc (Zn) (Figure 1F) and calcium (Ca) (Figure 1G), but not iron (Fe) and potassium (K) (data not shown) as compared to the control plants. In contrast, the growth and yield indicators remained unaltered when maize seeds were treated with nCu at concentration of 1000 mg/kg DW, indicating that the dose of nCu at 20 mg/kg DW could stimulate the growth and development of maize plants and improve crop yield.

***Morphological and pathological changes in mice fed with grain maize derived from nCu-treated seeds***

**Figure 2:**
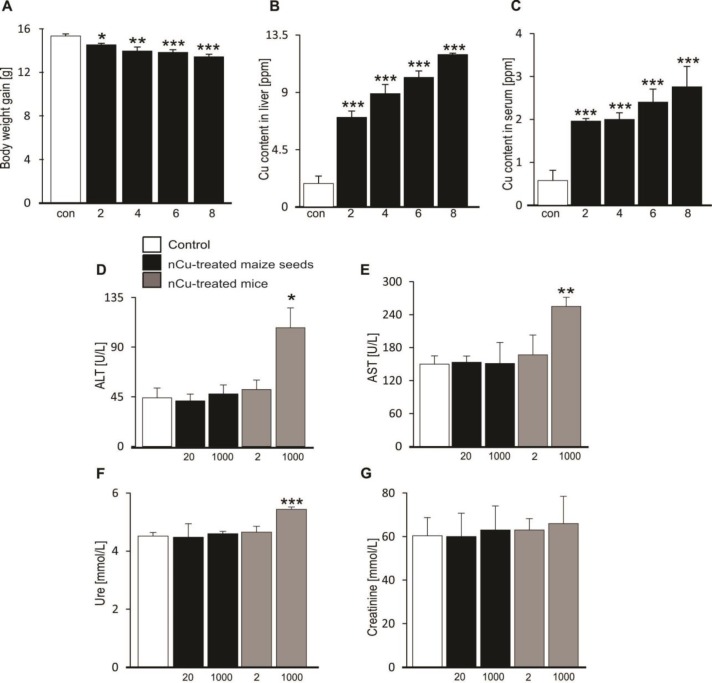
Effect of nano-copper on morphological and pathological changes in mice

To examine the morphological changes, all the mice were weighed every two weeks. As shown in Figure 2A, the average BW gain of the 1000 mg nCu/l-treated mouse group was not only elevated, but also significantly declined as compared to control group during the 8 weeks treatment, whereas changes in BW gain of the other four mouse groups including nCu-20, nCu-1000, and 2 mg nCu/l-treated mice as well as control were similar to each other (data not shown). Consequently, nCu treatment at dose of 1000 mg/l resulted in average weight loss of mice.

To determine factors causing the loss in BW gain, next experiments were performed to measure Cu deposition in liver tissues and serum of mice by the ICP-MS method. It is believed that physiological tolerance to excess Cu in mice might be important causes of a variety of diseases (2-5). Similar to changes in BW gain, we also observed that there was no significant differences in Cu content identified in serum and liver organ among the four mouse groups including nCu-20, nCu-1000, or 2 mg nCu/l-treated animals or control group at 2, 4, 6 and 8 weeks after modelling (data not shown). However, the mouse group supplemented with 1000 mg nCu/l had significantly increased storage of Cu concentration in both liver organ (Figure 2B) and serum (Figure 2C) as compared to control group, illustrating that the drinking water supplement with 1000 mg nCu/l caused the excessive accumulation of Cu in these systemic organs.

To assess a possible association between excessive Cu content and serum biochemical indicators of liver and kidney damages, additional experiments were performed to measure serum levels of AST, ALT, creatinine and urea of the 5 mouse groups. To be consistent with previous studies (1, 2), the 1000 mg nCu/l-treated mice had damaged functions of liver and kidney as denoted by significantly higher serum ALT (Figure 2D), AST (Figure 2E) and urea (Figure 2F) levels than that of control group, whereas no difference in serum creatinine level among the 5 groups was found (Figure 2G). The evidences pointed out that excessive Cu storage damaged the functions of liver and kidney in mice.

***Immune cell infiltration into spleens of mice fed with grain maize derived from nCu-treated seeds***

**Figure 3:**
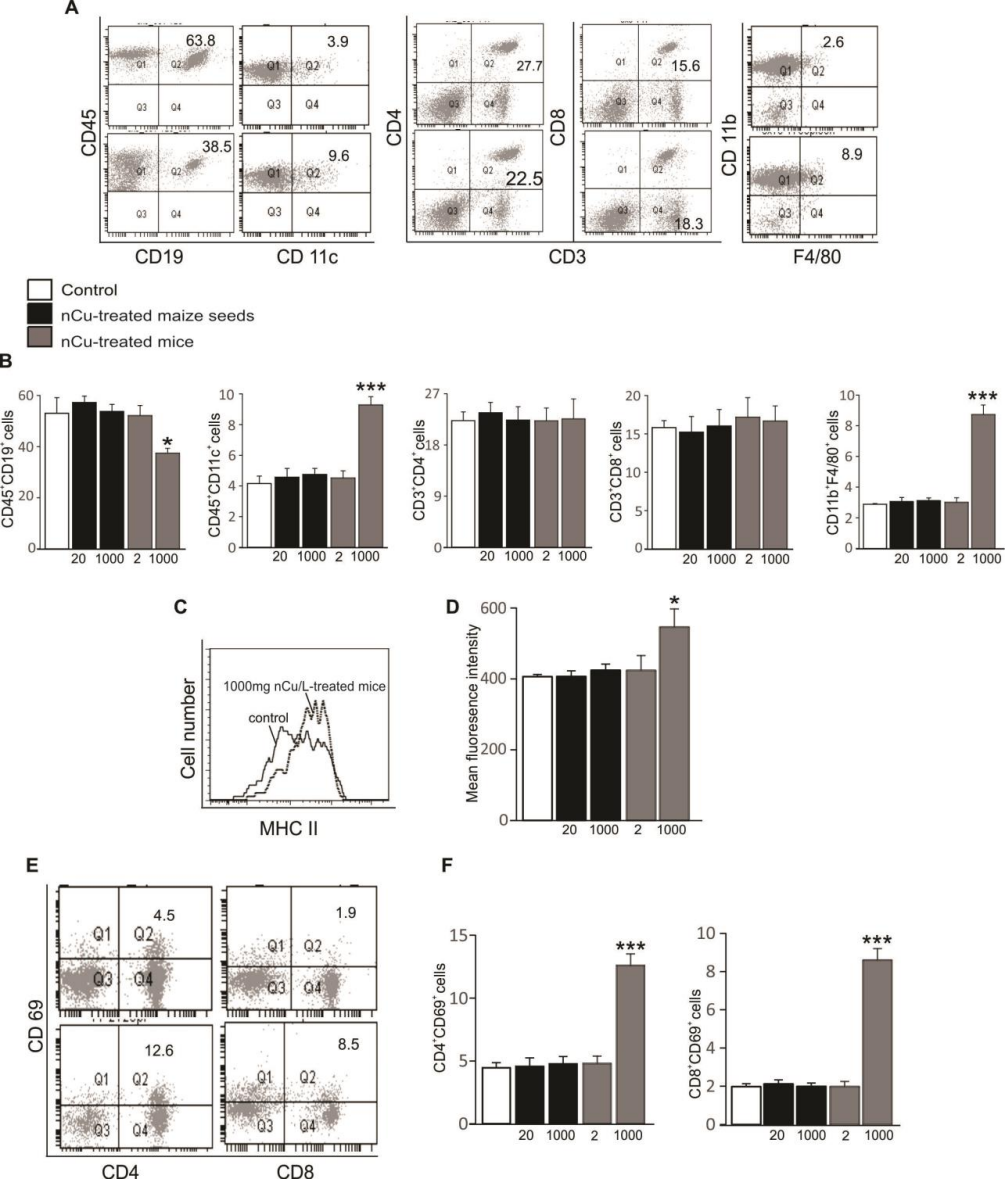
Effect of nano-copper on immune cell infiltration into mouse spleens

In addition to the signs of liver and kidney damages, recruitment of immune cells and their activation in tissues and organs reflects systemic inflammatory response in mice (11). Statistical analysis showed that mice supplemented with drinking water containing 1000 mg nCu/l displayed an increased infiltration of innate immune cells into spleens as well as an elevated activation of splenic leukocytes as compared to control group (Figure 3). In contrast, percentages of immune cells and their activation in spleens of the other mouse groups were unaltered (Figure 3). Accordingly, the numbers of antigen-presenting cells including dendritic cells (CD45^+^CD11c^+^) and macrophages (CD45^+^CD11b^+^F4/80^+^) infiltrated into spleens were high, whereas the number of B cells (CD45^+^CD19^+^) was found significantly lower in the 1000 mg nCu/l-treated mouse group than that in control group (Figure 3A-B). Consequently, increased expressions of major histocompatibility complex (MHC) class II molecule on CD45^+ ^cells (Figure 3C-D) as well as the upregulation of CD69 marker on both CD4 T cells (CD3^+^CD4^+^) and CD8 T cells (CD3^+^CD8^+^) (Figure 3E-F) were observed only in spleens of 1000 mg nCu/l-treated mice, but not in spleens of the other mouse groups. The evidences showed that the excessive accumulation of Cu in organs of the body such as liver and serum induced the infiltration of antigen-presenting cells and stimulated immune cell activation in mouse spleens.

***The*** ***apoptotic death of splenic leukocytes in mice fed with grain maize derived from nCu-treated seeds***

**Figure 4:**
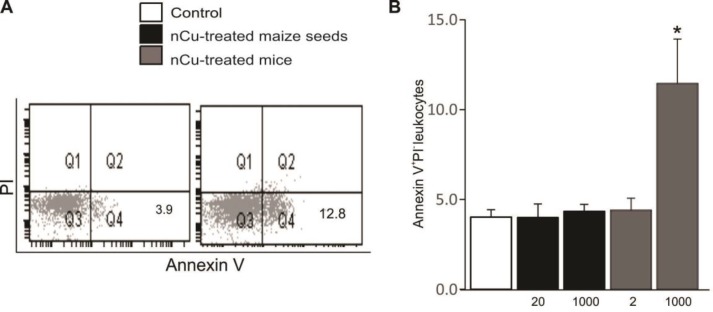
Effect of nano-copper on apoptotic death of splenic leukocytes in mice

The activation of immune cells results in programmed cell death; therefore, splenic leukocytes were examined for the apoptotic and necrotic cell death. A hallmark of apoptosis is cell membrane scrambling with subsequent PS exposure, whereas propidium iodide (PI) binding points to necrosis. As shown in Figure 4A-B, treatment of mice with 1000 mg nCu/l led to enhanced percentage of apoptotic leukocytes (Annexin V^+^/PI^-^ CD45^+ ^cells) in mouse spleens as compared to control group, whereas splenic leukocytes of the other mouse groups showed unaltered number of viable cells.

A. Images of maize seedlings after 7 days of germination, which were left untreated (1st panel) or treated with nCu at dose of 20 mg/kg dry weight (DW) (2nd panel) or 1000 mg/kg DW (3rd panel).

B. Arithmetic means (n = 3) of the germination percentage of maize seeds after 7 days, which were left untreated (control, white bar) or treated with nCu at dose of 20 mg/kg DW (black bar) or 1000 mg/kg DW (grey bar). ** (P<0.01) represents significant difference from control group, ANOVA.

C. Images of maize plants after 90 days of planting, which were left untreated (1st panel) or treated with nCu at dose of 20 mg/kg DW (2nd panel) or 1000 mg/kg DW (3rd panel)

D. Arithmetic means (n = 3) of maize grain yield, which were left untreated (control, white bar) or treated with nCu at dose of 20 mg/kg DW (black bar) or 1000 mg/kg DW (grey bar). *** (*P*<0.001) represents significant difference from control group, ANOVA.

E-G. Arithmetic means (n = 3) of Cu (E), Zn (F) and Ca (G) concentrations in maize grain, which were left untreated (control, white bar) or treated with nCu at dose of 20 mg/kg DW (black bar) or 1000 mg/kg DW (grey bar). * (*P*<0.05), ** (*P*<0.01) and *** (*P*<0.001) represent significant differences from control group, ANOVA

A. Arithmetic means (n=4-5) of body weight gain in mice, which were left untreated (control, white bar) or treated with 1000 mg/l nCu (black bar) for indicated time intervals (2, 4, 6 and 8 weeks). * (*P*<0.05), ** (*P*<0.01) and *** (*P*<0.001) represent significant differences from control group, ANOVA

B-C. Arithmetic means (n=4-5) of Cu content in liver (B) and serum (C) of mice, which were left untreated (control, white bar) or treated with 1000 mg/l nCu (black bar) for indicated time intervals (2, 4, 6 and 8 weeks). *** (*P*<0.001) represents significant difference from control group, ANOVA.

D-G. Arithmetic means (n=4-5) of serum alanine transaminase (ALT) (D), aspartate transaminase (AST) (E), urea (F) and creatinine (G) concentrations in mice, which were left untreated (control, white bars) or fed with grain maize derived from 20 mg/kg dry weight (DW) or 1000 mg/kg DW nCu-treated seeds (black bars) or treated with nCu at dose of 2 mg/l or 1000 mg/l (grey bars). * (*P*<0.05), ** (*P*<0.01) and *** (*P*<0.001) represent significant differences from control group, ANOVA

A. Original dot plots of CD45+CD19+, CD45+CD11c+, CD45+CD3+ CD4+, CD45+CD3+ CD8+ and CD45+CD11b+ F4/80+ cells recruited to spleens of mice, which were untreated (control, upper panels) and treated with 1000 mg/l nCu (lower panels). Numbers depict the percent of cells in the respective quadrants, acquired within the dead cell gate

B. Arithmetic means (n=4-5) of percentages of CD45+CD19+, CD45+CD11c+, CD45+CD3+ CD4+, CD45+CD3+ CD8+ and CD45+CD11b+ F4/80+ cells recruited to spleens of mice, which were left untreated (control, white bars) or fed with grain maize derived from 20 mg/kg dry weight (DW) or 1000 mg/kg DW nCu-treated seeds (black bars) or treated with nCu at dose of 2 mg/l or 1000 mg/l (grey bars). * (*P*<0.05) and *** (*P*<0.001) represent significant differences from control group, ANOVA

C. Histogram of major histocompatibility complex (MHC) class II expression was obtained by FACS analysis in a representative experiment on control (solid line) and 1000 mg nCu/l-treated splenic leukocytes (dotted line)

D. Arithmetic means ± SEM (n=5) of mean fluorescence intensity (MFI) of splenic leukocytes in mice, which were left untreated (control, white bars) or fed with grain maize derived from 20 mg/kg DW or 1000 mg/kg DW nCu-treated seeds (black bars) or treated with nCu at dose of 2 mg/l or 1000 mg/l (grey bars). * (*P*<0.05) represents significant difference from control group, ANOVA

E. Original dot plots of CD45+CD4+ CD69+ and CD45+CD8+ CD69+ cells recruited to spleens of mice, which were untreated (control, upper panels) and treated with 1000 mg/l nCu (lower panels). Numbers depict the percent of cells in the respective quadrants, acquired within the dead cell gate

F. Arithmetic means (n=5) of percentages of CD45+CD4+ CD69+ and CD45+CD8+ CD69+ cells recruited to spleens of mice, which were left untreated (control, white bars) or fed with grain maize derived from 20 mg/kg DW or 1000 mg/kg DW nCu-treated seeds (black bars) or treated with nCu at dose of 2 mg/l or 1000 mg/l (grey bars). *** (*P*<0.001) represents significant difference from control group, ANOVA

A. Original dot plots of Annexin V+ propidium iodide (PI)- leukocytes recruited to spleens of mice, which were untreated (control, left panel) and treated with 1000 mg/l nCu (right panel). Numbers depict the percent of cells in the respective quadrants, acquired within the dead cell gate

B. Arithmetic means (n=5) of percentages of Annexin V+ PI- leukocytes recruited to spleens of mice, which were left untreated (control, white bars) or fed with grain maize derived from 20 mg/kg dry weight (DW) or 1000 mg/kg DW nCu-treated seeds (black bars) or treated with nCu at dose of 2 mg/l or 1000 mg/l (grey bars). * (*P*<0.05) represents significant difference from control group, ANOVA

## Discussion

In this study, we indicated that maize grains obtained from the seeds treated with nCu at doses of 20 and 1000 mg/kg DW were safe to feed mice. The mice fed with the maize grains exhibited unaltered morphological and functional properties. Importantly, excessive deposition of hepatic and serum Cu of these mice were not detected, and further parameters including BW gain, hepatic and renal biochemical markers and percentages of infiltrated and active splenic immune cells were also found to be similar to the nCu-20, nCu-1000 and control groups. Interestingly, maize seeds treated with nCu at concentration of 20 mg/kg DW stimulated seed germination and plant growth as well as enhanced grain yield and grain mineral nutrients including zinc and calcium, whereas nCu treatment of seeds at concentration of 1000 mg/kg DW unaltered the physiological parameters of maize plants as compared to control plants. A recent study showed that plant tolerance to excess Cu is mediated through activities of antioxidant enzymes against ROS and Cu binding to the cell wall to induce exudates secretion (20), thus the concentration of nCu at 1000 mg/kg DW would be a tolerance threshold of maize plants. Differently, Cu treatment with dose of 8.0 mM inhibits seed germination and growth of wheat and cucumber plants (26), whereas soils supplemented with 250 mg Cu/kg soil do not affect the growth and development of maize plants due to the capability of the plants to inactivate the free Cu though metal chelation by high-affinity ligands (20). The tolerance mechanisms of plants against metals are high risk factors for human health when these plants are incorporated into the food chain (26). Consequently, treatment of maize seeds with 20 mg nCu/kg DW increased grain yield as well as mineral nutrients in maize, which was safe for mouse feed.

In addition to the promoting effect on plant morphological and physiological responses, nCu is essential for animal nutrition. Several studies have revealed that the dose of 2 mg nCu/l is a threshold limit value added to drinking water of mice [2, 3], although administration of intestinal epithelial cells with 2 mg nCu/l results in cell injury and toxicity (27). Differently, nCu treatment of chicken at the dose of 5 to 15 mg/l in drinking water has no effect on Cu content in the blood plasma (8). Our results denoted that supplementation with 2 mg nCu/l in the drinking water did not show any apparent changes in mice as proved by unaltered parameters including accumulation of Cu in liver and blood circulation, hepatic and renal biochemical markers and infiltration and activation of splenic immune cells as compared to the control group; therefore, nCu concentration of 2 mg/lL would be used for further applicable biomedicine. 

An imbalance in the supply of Cu relates to pathogenesis of multiple severe diseases including inflammation-related disorders (1, 3, 5, 6) and cell death via oxidative stress-dependent signalling cascades (28). In this study, we indicated further systemic pro-inflammatory and toxicological effects of nCu on kidney, liver and spleen of experimental mice. The inflammatory response related to Cu over-accumulation in systemic organs is characterized by local recruitment and activation of inflammatory leukocytes and intrinsic cell apoptosis (2, 10, 11, 17, 18). It is again consistent with our finding that pro-inflammatory and pro-apoptotic effects induced by excessive Cu storage in mice dosed with 1000 mg nCu /l were detected in splenic tissue as evidenced by infiltrations of macrophages and dendritic cells and increased activation of T lymphocytes. In addition, the serum levels of biochemical markers including ALT, AST and creatinine in 1000 mg nCu/l-treated mice were restored more than those in the control mice that reflected hepatic and renal defects in the 1000 mg nCu/l-treated mice. Moreover, we observed the increased PS exposure on splenic leukocyte surface in mice supplemented with drinking water containing 1000 mg nCu/l. The evidences indicated that nCu deposition caused hepatic and renal failures and induced inflammatory response in mice.

The pathogenic mechanisms associated with excessive Cu deposition and hepatic and renal damages are little known (10, 18, 28, 29). In this study, the splenic infiltrations of dendritic cells and macrophages, and antigen-presenting cells to primes naïve T cells in adaptive immune response that were exposed to nCu in inflamed tissues might cause activations of leukocytes including CD4 and CD8 T lymphocytes, leading to immune cell apoptosis and functional defects in systemic organs of mice.

## Conclusion

Treatment of maize seeds with nCu at dose of 20 mg/kg DW facilitated the morphological and functional development of maize plants; however, its high dose at 1000 mg/l in drinking water caused Cu accumulation in liver and blood circulation and could be the cause of autoimmune response as well as liver and kidney injuries in mice.

## Ethical approval

Animal care and experimental procedures were performed according to the Vietnamese law for the welfare of animals and were approved by the ethical committee of Institute of Genome Research.

## Conflicts of Interest

The authors of this paper declare that they have no financial/commercial conflicts of interests.
